# Isolation of Native Arbuscular Mycorrhizal Fungi within Young Thalli of the Liverwort *Marchantia paleacea*

**DOI:** 10.3390/plants8060142

**Published:** 2019-05-30

**Authors:** Yoshihiro Kobae, Ryo Ohtomo, Sho Morimoto, Daiki Sato, Tomomi Nakagawa, Norikuni Oka, Shusei Sato

**Affiliations:** 1 Laboratory of Crop Nutrition, Department of Sustainable Agriculture, Rakuno Gakuen University, Ebetsu, Hokkaido 069-8501, Japan; satod@docomo.ne.jp; 2 Hokkaido Agricultural Research Center, National Agriculture and Food Research Organization (NARO), 1 Hitsujigaoka, Toyohira, Sapporo, Hokkaido 062-8555, Japan; rotm@affrc.go.jp (R.O.); shomo@affrc.go.jp (S.M.); okan@affrc.go.jp (N.O.); 3 Central Region Agricultural Research Center, NARO, Kannondai 2-1-18, Tsukuba 305-8666, Japan; 4 NARO Headquarters, Kannondai 3-1-1, Tsukuba 305-8517, Japan; 5 Division of Symbiotic Systems, National Institute for Basic Biology, Nishigonaka 38, Myodaiji, Okazaki 444-8585, Aichi, Japan; nkgwtmm@nibb.ac.jp; 6 Graduate School of Life Sciences, Tohoku University, Katahira 2-1-1, Aoba-Ku, Sendai 980-8577, Japan; shuseis@ige.tohoku.ac.jp

**Keywords:** liverwort, *Marchantia paleacea*, mycothalli, native arbuscular mycorrhizal fungi, Sanger sequencing

## Abstract

Arbuscular mycorrhizal fungi (AMF) are a group of soil microorganisms that establish symbioses with most land plant species. “Root trap culture” generally has been used for isolating a single regenerated spore in order to establish a monospecific, native AMF line. Roots may be co-colonized with multiple AMF species; however, only a small portion of AMF within roots sporulate, and do so only under certain conditions. In this study, we tested whether young thalli (<2 mm) of the liverwort *Marchantia paleacea* harbour monospecific AMF, and can be used as a vegetative inoculant line. When *M. paleacea* gemmae were co-cultivated with roots obtained from the field, the young thalli were infected by AMF via rhizoids and formed arbuscules after 18 days post-sowing. Ribosomal DNA sequencing of the AMF-colonized thalli (mycothalli) revealed that they harboured phylogenetically diverse AMF; however, new gemmae sown around transplanted mycothalli showed evidence of colonization from phylogenetically uniform *Rhizophagus* species. Of note, mycothalli can also be used as an inoculum. These results suggest that the young thalli of *M. paleacea* can potentially isolate monospecific AMF from field soil in a spore-independent manner.

## 1. Introduction

The mutualistic relationship established by mycorrhizal fungi has a substantial impact on the nutrition, growth, and productivity of host plants [1,2,3]. Approximately 10% of vascular plant species, mostly woody species, are colonized by ectomycorrhizal fungi, which belong to mainly the *Basidiomycota* and *Ascomycota*, and the fungal hyphae grow extracellularly, forming a Hartig net and mantle [4,5]. Most of the remaining mycorrhizal fungi, with the exception of the ericoid- or orchid-specific mycorrhizal fungi, colonize nonwoody plant species and belong to the sub-phylum *Glomeromycotina*. This fungal group is generally known as arbuscular mycorrhizal fungi (AMF) [6], which encompass approximately 312 described species (http://www.amf-phylogeny.com/amphylo_species.html). AMF form highly branched hyphal structures, known as arbuscules, in root cortical cells, and spread intercellularly (*Arum*-type) or intracellularly (*Paris*-type) [7]. The formation of arbuscules has been regarded as the unique morphological feature of this symbiosis that is responsible for the nutrient exchange between the host plant and the AMF [8]. Laboratory-scale pot culture experiments involving the inoculation of a plant with one or more AMF isolates has established the mutual beneficial relationships [9]. A large number of studies have demonstrated that, in many plants, AMF colonization is followed by considerable stimulation of growth, and those plants contain a higher concentration of phosphate in their tissues than uncolonized controls [9]. Supporting this, in mutant plants carrying a deficient allele of symbiotic phosphate transporter genes, an abnormally early degradation of the arbuscules and a reduction in the total phosphate uptake and fungal biomass is observed [10,11,12]. This symbiosis is thought to have emerged more than 410 million years ago [13], and is considered an integral part of land plant evolution [6,14,15].

Differences and similarities in ribosomal DNA (rDNA) sequences are generally accepted as a reflection of the phylogenetic relationship between the AMF genus and species. The rDNA sequences detected from roots suggest the presence of more than one species from each genus within a single root [16], and different genera may coexist in root fragments as small as 1 cm in length [17]. Inoculation tests have been used to extrapolate the functional relationships within roots. However, rDNA sequencing of AMF mycorrhizas has shown evidence of the coexistence of diverse species of the genus *Glomus* species in bluebell (*Hyacinthoides nonscripta* L.) roots, using family-specific primers, but found very few *Glomus* spores within the associated rhizosphere [18]. This indicates that the majority of AMF colonize roots in a vegetative manner [19,20], and that their functionality cannot be assessed by inoculation studies in a spore-dependent manner. Thus, it would be beneficial to generate a vegetative, spore-independent AMF inoculum for the assessment and deeper understanding of the diverse AMF species and their functionalities.

AMF spores are generally used for the isolation, taxonomy, and preparation of inoculum [21]. However, root fragments can also serve as an alternative source of inoculum [22]; it has been shown that up to three AMF isolates of different morphotypes could be cultivated monoxenically from 4–5 mm long root fragments collected from the field [23]. Thus, shredded root fragments may be used for the establishment of monospecific AMF cultures in a spore-independent manner. However, the maintenance of a monoxenic culture system is time-consuming and expensive. Pot culture requires a large amount of space and a long cultivation period before monospecificity confirmation. As such, to the best of our knowledge, neither root fragments nor any mycorrhizal tissues other than spores have been determined suitable for the establishment of monospecific AMF derived from field samples.

Considering that roots inevitably host numerous AMF species, a smaller plant size (i.e., a lower cell number for colonization) may be more suitable for the isolation of a single AMF. Liverwort gemmae are <1 mm in size, and are abundantly produced in the gemmae cup on the thalli. If the gemmae (i.e., young thalli) can host AMF, then the earliest AMF colonization stage of the thalli sown on the soil is expected to contain a single native AMF individual. In this study, we tested whether the liverwort (*Marchantia paleacea*) can be used for the isolation of monospecific native AMF from fields. Isolation was performed in two steps. First, AMFs were isolated with young thalli from field roots that were colonized with multiple native AMFs (first trapping). Second, AMF from the first trapping thalli were further isolated with new young thalli (second trapping). In addition, we tested whether AMF-colonized thalli (i.e., “mycothalli” [24]) can feasibly inoculate other plants.

## 2. Results and Discussion

### 2.1. Growth Condition of M. paleacea Young Thalli

A large thallus with numerous rhizoids and thick parenchyma cells can potentially harbour multiple AMF, and thus hamper the isolation of individual AMF. To establish a method for the isolation of individual AMF, we investigated the timing that would allow small thalli to be colonized by only a few AMF individuals at the early colonization stage. In the preliminary trials for setting up cultivation conditions enabling young *M. paleacea* thalli to be rapidly and uniformly colonized by AMF, we found that brightly-coloured soils (e.g., brownish volcanic lapillus) enabled the thalli to grow up so as to leave from the soil (i.e., warp), which eventually inhibited uniform AMF colonization. We encountered another problem that needed to be overcome for the subsequent cytological and molecular analysis of young mycothalli, in that soil granules must be removed as much as possible from the rhizoids. In typical field soils, numerous rhizoids extended several millimetres deep and adhered tightly to the soil matrix, hampering subsequent AMF staining and DNA sequencing procedures. Thus, it is important to prevent rhizoids from adhering to the soil matrix, in order to accurately evaluate the colonization level. To overcome these pitfalls, we employed the use of an artificial aquarium soil, which is black, granular, compacted, indigenous AMF-free, and generated from andosols. Andosols originate from volcanic ash soil and have high phosphate absorption potential [25], thus preventing the occurrence of phosphate inhibition that may decrease AMF colonization [26,27,28]. These compacted soil granules rarely collapse when gripped with soft tip tweezers, and were therefore easily removed from rhizoids.

### 2.2. Young Thalli (<2 mm) Colonized by Arbuscular Mycorrhizal Fungi

To assess the potential of *M. paleacea* thalli to be colonized with diverse AMF species, the *M. paleacea* gemmae were co-cultivated with AMF culture strains (*Claroideoglomus etunicatum*, *Rhizophagus intraradices*, *Gigaspora margaria*, and *Ambispora callosa*). All AMF strains formed arbuscules in the parenchyma cells of the mycothalli, and *C. etunicatum* and *R. intraradices* formed vesicles at 25 days post-sowing (dps) (Figure S1), suggesting successful colonization. To assess the timing of the earliest AMF colonization, young thalli were inoculated with a model AMF (*R. irregularis* DAOM197198). The hyphae of *R. irregularis* infected thalli intracellularly via rhizoids and formed arbuscules in the thallus parenchyma cells at 18 dps (Figure S2), while arbuscule formation was not observed until 14 dps. Thalli with arbuscules exhibited dark red pigmentation in the central midrib area (Figure S3A), as previously reported [29,30]. Notably, thalli lacking arbuscules, but with only hyphal colonization or no colonization in the rhizoids, showed light red pigmentation at the central midrib area in some cases (Figure S3B). This red pigmentation was not observed in the thalli of AMF-free controls, suggesting that red pigmentation can occur at the pre-symbiotic stage. Based on the timing of this dark red pigmentation and the colonization level, the earliest colonization stage with only a few AMF infections was determined to be 18 dps, at least within the AMF model of *R. irregularis*; accordingly, the mycothalli rDNA of native AMF were analysed at this stage.

### 2.3. First Trapping of Arbuscular Mycorrhizal Fungi and Sequencing

**Figure 1:**
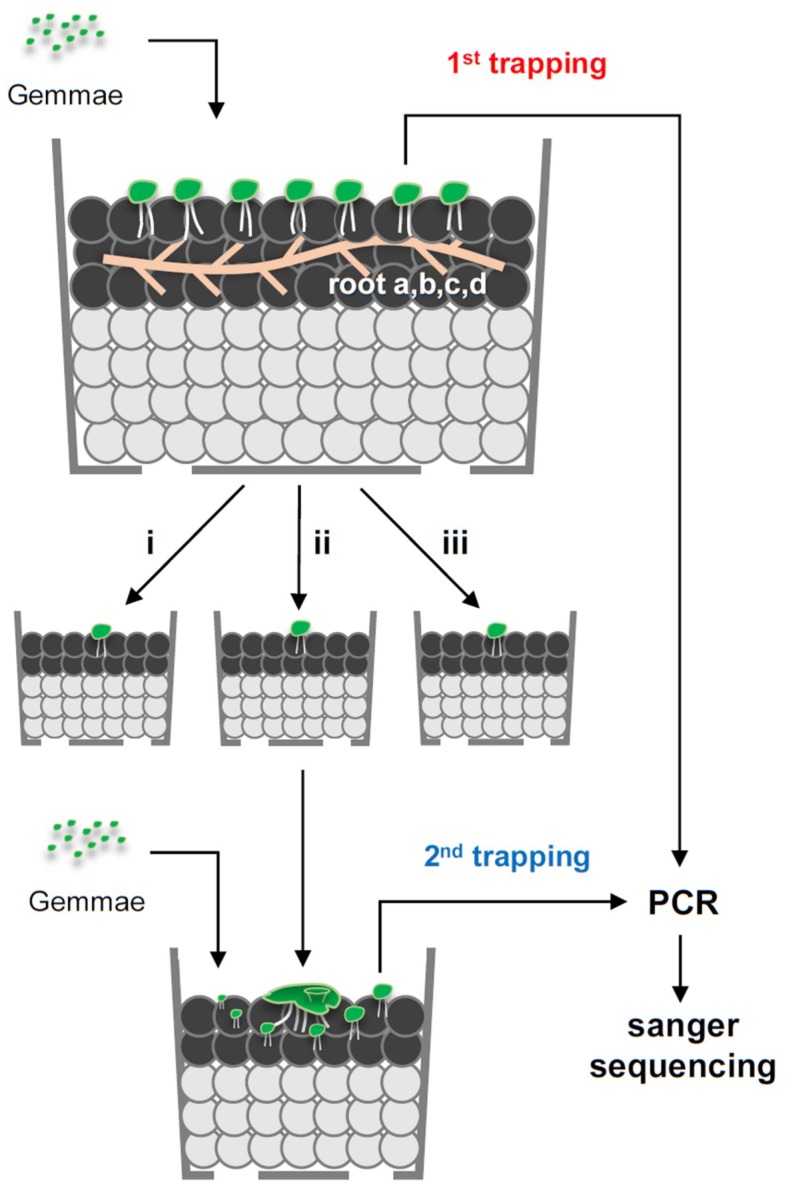
Trapping/isolation of native arbuscular mycorrhizal fungi (AMF) and DNA sequencing within liverwort. Schematic representation of the cultivation system of mycorrhizal *Marchantia paleacea* and AMF rDNA sequencing. *M. paleacea* gemmae harvested from gemmae cups were sown into soils where field roots (four types: a–d) were buried at a depth of ~5 mm. Gemmae (nascent thalli) were grown for 16–20 days, to allow colonization of native AMF from the roots. Three mycorrhizal thalli (mycothalli; i, ii, and iii) from the earliest colonization stage with red pigmentation in the central midrib area were transplanted to new pots. Three to ten mycothalli with red pigmentation were subjected to PCR and rDNA sequencing. *M. paleacea* gemmae harvested from the gemmae cups were sown around transplanted mycothalli (donor) for 20–30 days to allow colonization of the AMF from donor mycothalli. Six to eight mycothalli with red pigmentation per donor mycothalli were subjected to rDNA sequencing. DNA isolation, PCR, and Sanger sequencing were performed as previously described [31].

**Figure 2:**
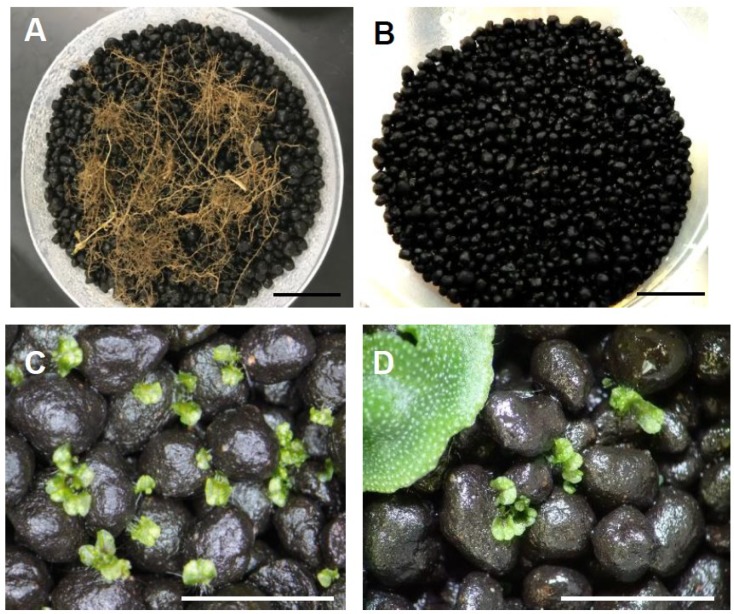
Pot preparation and mycothalli images. (**A**) Roots were deposited on soil. (**B**) Roots were buried within soils at a depth of ~5 mm. (**C**) Gemmae (young thalli) of *Marchantia paleacea* at 14 days post-sowing (dps) (i.e., first mycothalli). (**D**) *M. paleacea* gemmae at 14 dps (i.e., second mycothalli) were transplanted around donor mycothalli. Bar = 2 cm (**A**,**B**); 5 mm (**C**,**D**).

**Figure 3:**
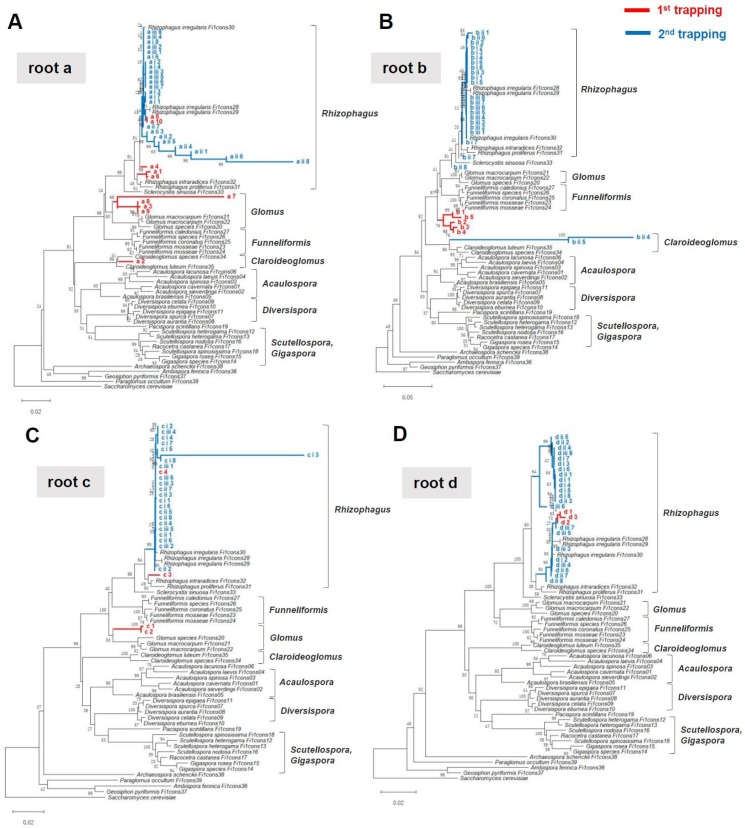
Phylogenetic placement of AMF rDNA mycothalli partial sequences. For phylogenetic tree construction, the first mycothalli “meta”-sequences (*n* = 3–10, shown by red lines) and second meta-sequences (*n* = 6–8, shown by blue lines) from different roots, 39 AMF rDNA consensus sequences [32], and the *Saccharomyces cerevisiae* rDNA sequence were used. The *S. cerevisiae* sequence was used as the outgroup. **A**–**D** show the analyses for roots a, b, c, and d, respectively. Phylogenetic trees were generated using the neighbour-joining method. The percentage of replicate trees in which the associated taxa clustered together in the bootstrap test (i.e., 500 replicates) are shown next to the branches. Evolutionary distances were computed using the maximum composite likelihood method. Evolutionary analyses were conducted in MEGA X [33].

Native AMF isolation was carried out in two steps. First, *M. paleacea* gemmae were co-cultivated with roots that were buried within depths of about 5 mm (Figure 1 and Figure 2). Young thalli with dark red pigmentation were randomly selected, and individual rDNA sequences were obtained by Sanger sequencing (first trapping, see Figure 1). We used the universal fungal primer set LR1-FLR2 to PCR amplify the rDNA of diverse AMF species. However, BLAST analysis showed that these primers targeted the rDNA sequences of *M. paleacea*. Thus, PCR was subsequently performed with AML1–AML2 AMF-specific primers. We amplified the partial rDNA of mycothalli (*n* = 4–10) that were co-cultivated with roots obtained from four different vegetation types (23 mycothalli in total); agarose gel electrophoresis of these PCR products produced primarily single bands of the expected size (approximately 780 bp). Sanger sequencing of these PCR products with AML1 primers and the BLAST analysis results indicated that 22 of the sequences belonged to Glomeromycotina, with one sequence not being analysed due to a low signal. Phylogenetic placement of these sequences suggested that each of the mycothalli are potentially colonized with different AMF species (e.g., *Rhizophagus*, *Funneliformis*, *Glomus*, and *Claroideoglomus*) (first trapping, see Figure 3).

### 2.4. “Meta”-Sequences of Arbuscular Mycorrhizal Fungi rDNA in Young Thalli

Electropherograms of these sequences showed that some mycothalli were potentially colonized with a monogenic AMF, based on the base calling purity (i.e., quality value) (Figure S4B); however, other mycothalli showed a mixed electropherogram (Figure S4C). In a previous study, we investigated rDNA sequences of native AMF within an infection unit, each of which comprised an internal mycelium arising from one entry point [34,35] in the mycorrhial roots of rice (*Oryza sativa* L.) at very early colonization stages (8–12 days post-planting) [31]. Rice root segments (<3 mm) containing an infection unit were dissected and pulverised, large subunits of rDNA were amplified using universal fungal primers, and the sequences were directly determined by Sanger sequencing. All obtained sequences belonged to Glomeromycotina. However, infection unit-based sequencing revealed the presence of inter-specific diversity in the heterogeneity of the rDNA sequences [31]. Supporting this, recent studies of single nucleus sequencing for some AMF culture lines demonstrated that these are not only homokaryons, but also dikaryons and heterokryons [36,37]. Furthermore, long-read whole genome sequencing of *R. irregularis* DAOM197198 indicates that the genome contains 10 different rDNA sequences that are differentially localized (i.e., non-tandem repeats) amongst the chromosome [38]. These findings suggest that a one-to-one relationship may not be applicable to the rDNA sequences and the genetic identities of the individuals. Accordingly, the rDNA sequences derived from mixed electropherograms are not the actual sequences, but instead illustrate the representative “meta”-sequences [31]. There is a possibility that some mycothalli with mixed electropherograms may contain monospecific AMF, while others contain multiple AMF.

### 2.5. Second Trapping Isolated Phylogenetically Uniform Arbuscular Mycorrhizal Fungi Species

To assess multiple colonizations of different AMF species within mycothalli, a second trapping was performed. The mycothalli isolated from the first trapping from each of the pots (donor, *n* = 3) were randomly selected and transplanted into new pots, and new gemmae (i.e., recipients) were sown around the donor mycothalli and co-cultivated for 20 days (Figure 2D). Then, recipient thalli with dark red pigmentation (*n* = 6–8) were randomly selected from each pot (93 mycothalli in total). All PCRs produced a single band, and rDNA meta-sequences were obtained for 91 mycothalli; two sequences were not obtained, due to a sequence’s low signal. Phylogenetic placement and BLAST analyses of these sequences indicated that, unexpectedly, all recipient mycothalli contained phylogenetically uniform *Rhizophagus* species (Figure 3), suggesting that cultivation or host bias narrowed AMF diversity within mycothalli. All mycothalli from the first trapping of root d harboured *Rhizophagus* species (Figure 3D), as did those of the second trapping. However, in the first trapping with roots a, b, and c, the isolated AMF species differed phylogenetically from those of the second trapping (Figure 3A–C). There are two possible explanations for these observations. Firstly, multiple AMF species could have rapidly colonized the young thalli of the first trapping by 18 dps. Amongst these, during the second trapping, *R. irregularis* dominantly colonized the recipient thalli. Mycothalli 3,3′-diaminobenzidine (DAB) staining at 18 dps revealed that some mycothalli contained multiple rhizoids that were infected by fungal hyphae. The soil environment of the first versus second trappings differed, as the former contained field materials (e.g., roots, microbes), and the latter only small mycothalli. The different environments might have altered the AMF species composition within the second isolation culture. A second possible scenario is that small thalli can only harbour a single AMF, but with genetically different nuclei or nucleotypes [39,40] that segregate, with some nuclei accommodating the thalli of the second trapping due to the differing environment. The AMF strain genotype can change during a host plant shift [41]. There is an AMF strain that harbours two co-existing nucleotypes that can undergo recombination [37]. Thus, the native AMF in this study might also shift its nucleotype between the first and second trapping, and eventually *R. irregularis* could virtually dominate the isolated mycothalli. Given that the coexistence of nucleotypes belonging to distinct AMF species has not been observed for any AMF strain, there is no evidence to support the latter scenario. However, intraspecific nucleotype alteration may occur in mycothalli between a first and subsequent isolation. Further study is needed to understand the genetic dynamics of native AMF within mycothalli.

### 2.6. Mycothalli Can Be Used as an Arbuscular Mycorrhizal Fungi Inoculum in Pot Experiments

**Figure 4:**
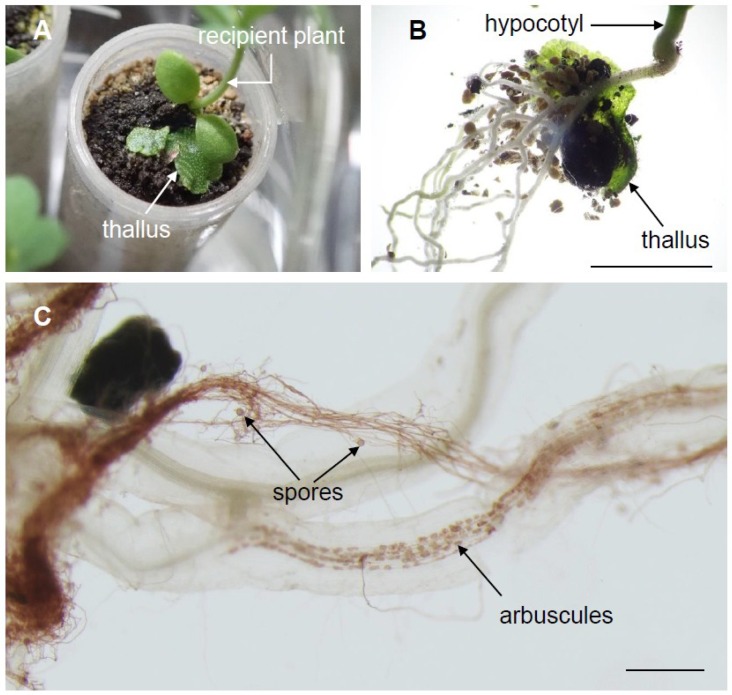
Mycorrhizal thalli as AMF inoculants. (**A**) Image of *Lotus japonicas* seedlings inoculated with mycorrhizal thalli (*Marchantia paleacea* colonized with native AMF), taken at 10 days post-inoculation (dpi). (**B**) Image of mycothalli adhered to *L. japonicus* root at 10 dpi. (**C**) Roots were stained with 3,3′-diaminobenzidine staining with horseradish peroxidase (HRP)-conjugated wheat germ agglutinin (WGA) (WGA–HRP–DAB) at 10 dpi. Bars = 5 mm (**B**), 0.5 mm (**C**).

To test whether native AMF trapped by individual thalli can be used for inoculating different plant species, mycothalli from a second trapping were co-cultivated with *Lotus japonicas* (Figure 4). Preliminary tests showed that mycothalli buried lower than 2 cm in the soil maintained their inoculation potential. We found that only sowing thalli next to a recipient plant was sufficient for rapid inoculation. In this case, the soil surface and mycothalli should be kept at high humidity conditions to prevent evaporation. Rhizoids stiffly attached to the bottom of the hypocotyl, and arbuscules were formed in the recipient roots at 10 dps (Figure 4B). Interestingly, bundles of AMF hyphae with spores were often observed (Figure 4C). Polar growth of the massive hyphal structure has not been reported, and rhizoids might guide the extension of AMF hyphae to roots. 

In conclusion, although our experiment was limited to a single growth condition with two transplantations, the potential applicability of this trapping method to isolate native AMF in a vegetative and spore-independent manner was demonstrated. For future studies, different growth conditions (e.g., soil nutrients and microbes, temperature, etc.) will allow isolation of more diverse AMF species that rarely sporulate. Furthermore, diverse plant roots obtained from various soil types would allow for the exploration of the existence of unknown AMF. In addition, it is possible that small liverwort thalli harbouring single AMF individuals will allow a deeper understanding of the genomic dynamics of diverse native AMF.

## 3. Materials and Methods

### 3.1. M. paleacea Thalli Growth Conditions

To maintain *M. paleacea* subspecies *diptera* [42], thalli were cultivated in polyethylene pots (80 mm in height; 90 mm maximum diameter; four holes of 6 mm in diameter at the bottom), containing 200 mL of Ezo sand (bottom layer; small pumice) and 30 mL of an artificial aquarium soil (upper layer; BB sand, GEX corporation, Osaka, Japan), which is black, granular (approximately 2–3 mm in diameter), and fertilizer- and indigenous AMF-free (i.e., no colonization occurred within 1 month of cultivation without the inoculation of exogenous AMF). The top section of aquarium soil was immersed in excess tap water for 1 day prior to use, then rinsed and dried for 2 weeks at 40 °C. Diluted Murashige Skoog (MS) solution (0.05× strength, 100 mL per pot) was supplied at the bottom of the pot once at the initiation of cultivation. The pot was covered with a plastic petri dish lid (90 mm in diameter) to prevent evaporation. Thalli were maintained under controlled environmental conditions (26 °C with 16 h light/day). The thalli tips (0.5–1 cm) containing apical notches (i.e., marginal meristems) were cut once monthly and transplanted into a new pot.

### 3.2. Inoculation Test

For the inoculation test of the cultured AMF lines (*Rhizophagus irregularis* DAOM197198 (Premier Tech, Canada), *Claroideoglomus etunicatum* MAFF520053, *Rhizophagus intraradices* MAFF520059, *Gigaspora margaria* MAFF520074, and *Ambispora callosa* MAFF520084), the inoculant of each line (i.e., 500 spores or 1 g inoculum for *R. irregularis* DAOM197198 and MAFF lines, respectively) was placed separately below the upper soil in the same pot design used to maintain the thalli. Fresh gemmae were collected from gemmae cups and transferred to water containing 0.001% Triton-X100, after which they were sown on the upper soil (approximately 100 gemmae per pot). Pots were placed under controlled environmental conditions (26 °C with 16 h light/day).

### 3.3. Arbuscular Mycorrhizal Fungi Cell Wall Staining

The AMF cell walls were visualized with 3,3′-diaminobenzidine (DAB) staining [43]. Thalli (<2 mm) separated from soil granules were transferred to 2 mL centrifuge tubes and cleaned using 10% *w*/*v* potassium hydroxide at 80 °C in a preheated heat block for 8 min, then washed once with 0.5 mL methylene blue (0.1 mg/mL), five times with 1 mL water, and once with 1 mL phosphate-buffered saline (PBS; pH 7.5). Thalli were then immersed in 1 mL PBS containing 0.5% *w*/*v* skim milk (Wako, Osaka, Japan) and 0.4 μg mL^−1^ wheat germ agglutinin (WGA)-conjugated horseradish peroxidase (HRP; Vector, Burlingame, CA, United States). Thalli were incubated in this solution at room temperature for at least 8 h before being washed twice with 1 mL PBS, then were immersed in 1 mL PBS containing 0.2 mg mL^−1^ DAB tetrahydrochloride (Nakarai Tesque, Kyoto, Japan) and 0.1 μL mL^−1^ 30% H_2_O_2_. Thalli were incubated in the DAB solution for at least 30 min at room temperature, then were soaked in 1 mL Tris-ethylenediaminetetraacetic acid (Tris-EDTA) buffer (TE buffer; 10 mM Tris-HCl, 1 mM EDTA; pH 8.0) to stop the HRP reaction. Images were obtained using a stereomicroscope (SZX16 or SZ61, Olympus, Japan) equipped with a charge-coupled device camera.

### 3.4. Trap Culture

Four bulk roots (a–d, see Figure 1) were obtained from a field at Rakuno Gakuen University, Hokkaido, Japan. The root species were not identified. Roots a and b were collected from roadside vegetation (root a: 43°04’11.2” N, 141°30’29.6” E; root b: 43°04’18.1” N, 141°30’35.2” E). Root c was obtained from vegetation under a pine tree (43°04’17.4” N, 141°30’25.5” E). Root d was taken from a fallow field that was bare for >10 years (43°04’11.5” N, 141°30’29.9” E). Roots were severed from plants and rinsed with tap water to remove soil, then 3 g (fresh weight) were buried at a depth of about 5 mm in the upper aquarium soil in the pots described above (Figure 1 and Figure 2A,B). Fresh gemmae were collected from gemmae cups and transferred to water containing 0.001% Triton-X100, then sown on the upper soil (approximately 100 gemmae per pot).

For the first trapping, young thalli at 18 dps were sampled for analysis of colonization level, transplanting, and DNA sequencing. For transplanting, upper soil granules adhered to rhizoids were removed. In this stage, 1–5 soil granules adhered to the rhizoids of a single thallus. To assess thalli intactness during the removal of soil granules, thalli were stained with 0.1 mg/mL methylene blue solution for 5 min, then were rinsed with water. The rhizoid intactness was then assessed with a stereomicroscope. Rhizoids that adhered to soil tended to be wavy and flexible. While separating thalli from soil granules, rhizoids never fell out from the bases, but a portion (<10%) of long rhizoids were severed during the separation of mycothalli from soil granules. Despite this partial damage to rhizoids, there was no obvious impact on the subsequent growth of thalli after transplantation, compared to those with soil granules that remained fully attached. Thus, to decrease AMF contamination unrelated to mycothalli, soil granules were removed from thalli for transplantation by rinsing in water with soft tip tweezers.

For the second trapping, thalli from the first trapping were transplanted into new polystyrene pots (50 mm in height; 70 mm maximum diameter; four holes 6 mm in diameter at the bottom) containing 60 mL of Ezo sand (bottom layer) and 15 mL of aquarium soil (upper layer) (Figure 1). MS solution (0.05× strength, 20 mL per pot) was supplied from the bottom once at the initiation of cultivation. The pot was covered with a plastic lid to prevent evaporation. Thalli were cultivated as mentioned above.

### 3.5. PCR and Sequencing

AMF mycothalli rDNA analysis was performed as previously described, according to the procedure for single AMF (i.e., infection unit) DNA analysis from small root fragments (<3 mm in length) [31]. Thalli (<2 mm) separated from soil granules at 18–20 dps were placed in 12 μL of TE buffer on a coverslip (24 × 50 mm). The sample was then covered with another coverslip (18 × 18 mm), with care taken to avoid the formation of air bubbles between coverslips. Samples were carefully pressed between the coverslips with an eraser. Approximately 5 μL of sample solution pressed out from the upper coverslip was recovered, and 1 μL was used as the PCR template. KOD One PCR Master Mix -Blue- (TOYOBO, Osaka, Japan) was used for PCR, with the universal fungal primers LR1/FLR2 [17,44] or AMF-specific primers AML1/AML2 [45]. The reaction mix was prepared according to the manufacturer’s instructions. Thermal cycling was performed in a Biometra Tadvanced Twin 46G (Analytik Jena A, Jena, Germany), under the following conditions: 10 s of initial denaturation at 98 °C; 40 cycles with 10 s of denaturation at 98 °C, 5 s of annealing at 58 °C, and 1 s elongation at 68 °C; and a final extension phase of 10 s at 68 °C. The same PCR conditions were used for all primer sets. PCR products were separated by gel electrophoresis on a 1.0% agarose gel in TAE buffer (40 mM Tris acetate, 1 mM EDTA, pH 8.2), DNA was visualised with the fluorescent intercalator Midori Green Direct (Nippon Genetics, Tokyo, Japan) and excited by a Blue/Green LED illuminator (wave length approx. 500 nm) (Nippon Genetics, Tokyo, Japan). PCR products of the expected size were excised and extracted using the FastGene Gel/PCR Extraction kit (Nippon Genetics, Tokyo, Japan), then were sequenced with the LR1 or AML1 primers using an ABI 3730 capillary sequencer with BigDye v3.1 sequencing chemistry (Thermo Fisher Scientific). The sequence taxa obtained via PCR were verified using the National Center for Biotechnology Information’s BLAST. The sequences were assembled and edited in MEGA X software [33].

### 3.6. Sequence Analysis and Classification

The sequences of the obtained PCR products were entered into a BLAST search to verify the sequences taxa. Sequences—including isolated mycothalli sequences and AM fungal SSU-ITS-LSU reference data (http://www.amf-phylogeny.com; [32])—were aligned using the MUSCLE program (Edgar, 2004) on MEGA X [33]. The aligned sequences were shortened to lengths corresponding to the nucleotide region 386–1052 of *Rhizophagus irregularis* MUCL43195 (consensus 28) [32]. A phylogenetic tree was generated with MEGA X [33], using the maximum likelihood method with a Kimura two-parameter model plus gamma. *Saccharomyces cerevisiae* NRRL Y-12632 was used as an outgroup. The reliability of the phylogenetic tree clades was assessed using the bootstrap method, with 500 replications. Electropherograms were drawn using Sequence Scanner Software 2 (Applied Biosystems, http://www.appliedbiosystems.com).

### 3.7. Mycothalli Inoculation Test

To assess mycothalli inoculant capabilities, mycothalli were co-cultivated with *Lotus japonicas* MG-20 in 12 mL polypropylene tubes (90 mm in height; 17 mm in diameter; one hole 6 mm in diameter at the bottom), containing 4 mL of aquarium soil (bottom layer) covered by a mixture of calcined attapulgite clay and mixer-crashed granular potting soil (Kumiai, JA Hokuren, Hokkaido, Japan) (20:1 by weight; upper layer). A mycothallus (28 dps) was placed proximal to the *L. japonicas* seedling. No other nutrients were added except for those within the granular potting soil, and water was supplied from underneath. Tubes were covered with transparent plastic bags to prevent evaporation, because drought and salt accumulation at the soil surface, both of which are driven by evaporation, strongly inhibit mycothalli growth and delay AMF colonization. AMF colonization within *L. japonicas* roots was detected by DAB staining, as described previously [43].

## Supplementary Materials

The following are available online at https://www.mdpi.com/2223-7747/8/6/142/s1, Figure S1: Microscopic observation of young *Marchantia paleacea* thalli inoculated with various AMF species; Figure S2: Microscopic observation of AMF colonization in *Marchantia paleacea* thalli and schematic representation of the colonization processes; Figure S3: Different red pigmentation in the mycothalli of *Marchantia paleacea*; Figure S4: Sequence heterogeneity of the mycothalli rDNA Sanger sequences.
