# Supplementary material for: Isolation of Native Arbuscular Mycorrhizal Fungi within Young Thalli of the Liverwort Marchantia paleacea
Source: Plants (Basel). 2019 May 30;8(6):142. doi: 10.3390/plants8060142 (PMC6631804; doi:10.3390/plants8060142)
Supplement: Supplementary file 1 [file plants-08-00142-s001.zip › plants-500447-SI.pdf]

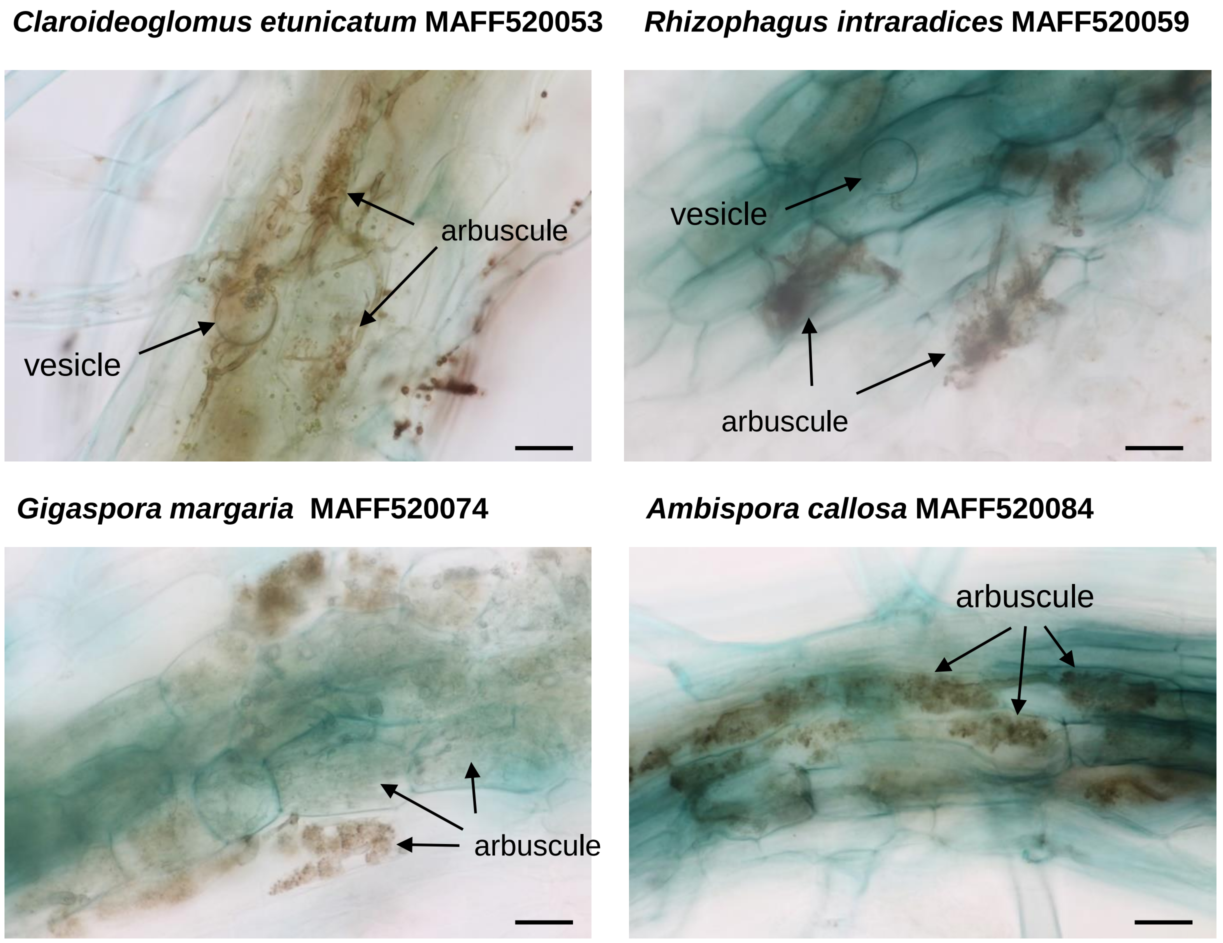

Supplementary data

Figure S1.

Microscopic observation of young *Marchantia paleacea* thalli inoculated with various AMF species. Bar = 20 µm.

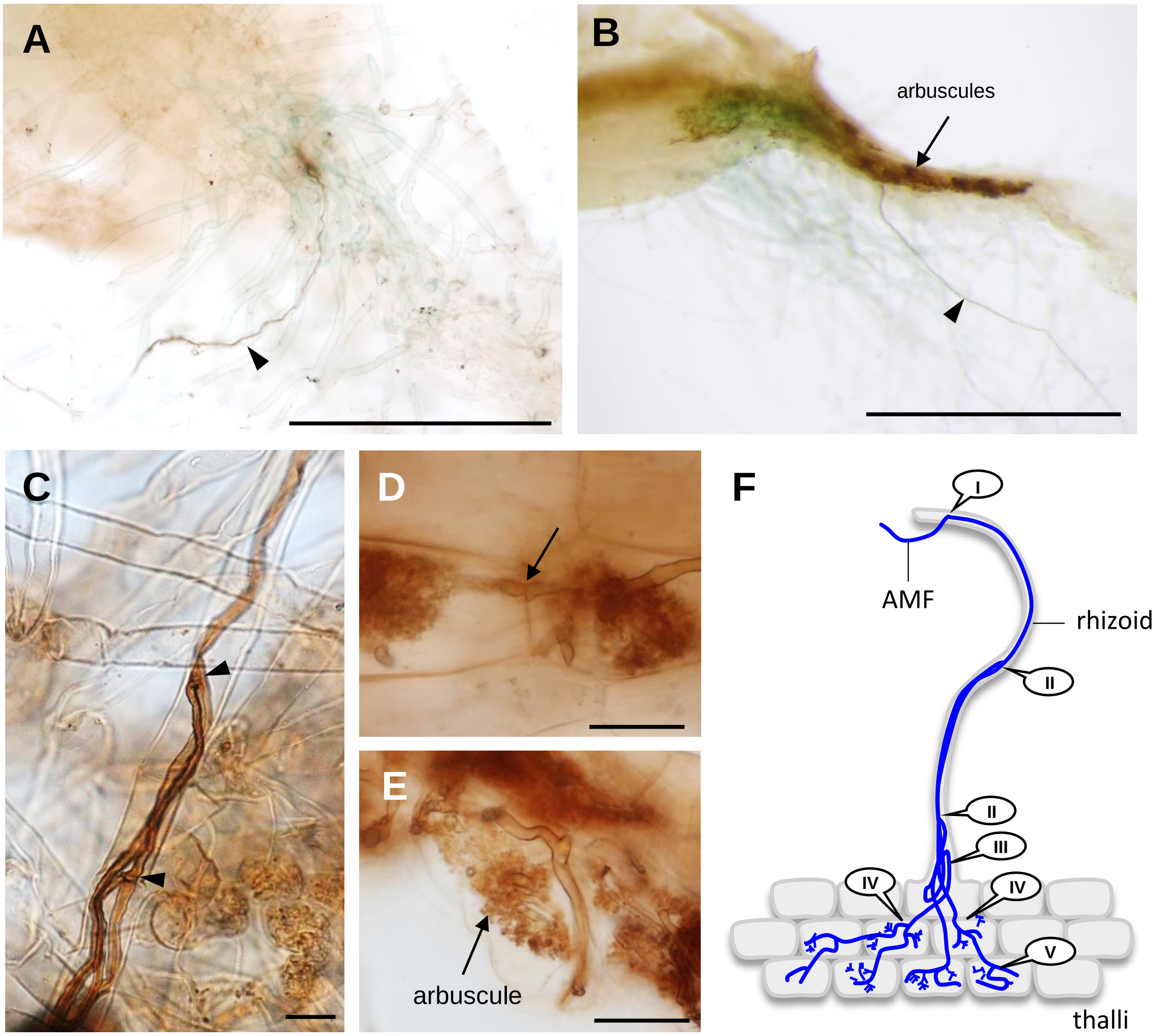

Supplementary data

Figure S2.

Microscopic observation of AMF colonisation in *Marchantia paleacea* thalli and schematic representation of the colonisation processes. The *M. paleacea* mycothalli (inoculated with *Rhizophagus irregularis* DAOM197198) were stained with WGA-HRP-DAB (3,3'-diaminobenzidine staining with HRP-conjugated wheat germ agglutinin). (**A**) Thallus of the earliest colonisation stage (18 dps). Thalli were stained with WGA-HRP-DAB. Arrowhead indicates infecting hyphae within rhizoid. The hyphae approached the base of rhizoid cells, and no colonisation is observed in parenchyma cells. (**B**) Thallus at 28 dps. Only a single infecting hyphae is observed, while arbuscules are formed in parenchyma cells. (**C**) Rhizoid colonized with hyphae. Arrowheads indicate the positions of hyphal branching within rhizoid. (**D**) Arrow indicates the position of cell-cell penetration of intracellular hypha. (**E**) Arbuscules develop in parenchymal cells. (**F**) Schematic presentation of the colonisation process at the early colonisation stage of *R. irregularis* DAOM197198 in the young *M. paleacea* thallus. During the earliest colonisation stage, hyphal colonisation is observed only in rhizoids of the ventral side of the thalli. First, hypha penetrates the rhizoid (I), then extends toward the thalli, branches several times (II), coils at the base of the rhizoid cell (III), penetrates into parenchymal cells (IV) and form arbuscules (V). Bar = 0.5 mm (**A**); 1 mm (**B**); 20 µm (**C**–**E**).

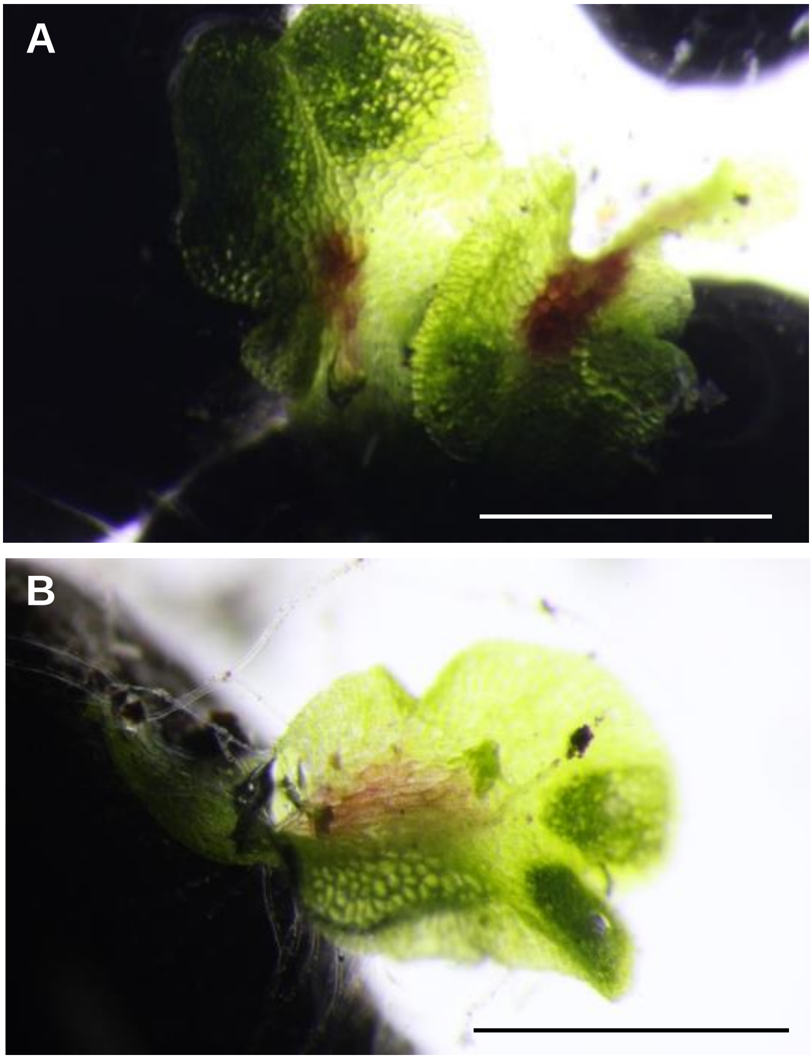

Supplementary data
Figure S3.
Different red pigmentation in the mycothalli of *Marchantia paleacea*. (**A**) Arbusculated mycothalli. (**B**) Mycothalli lacking arbuscules. Bar = 1 mm.

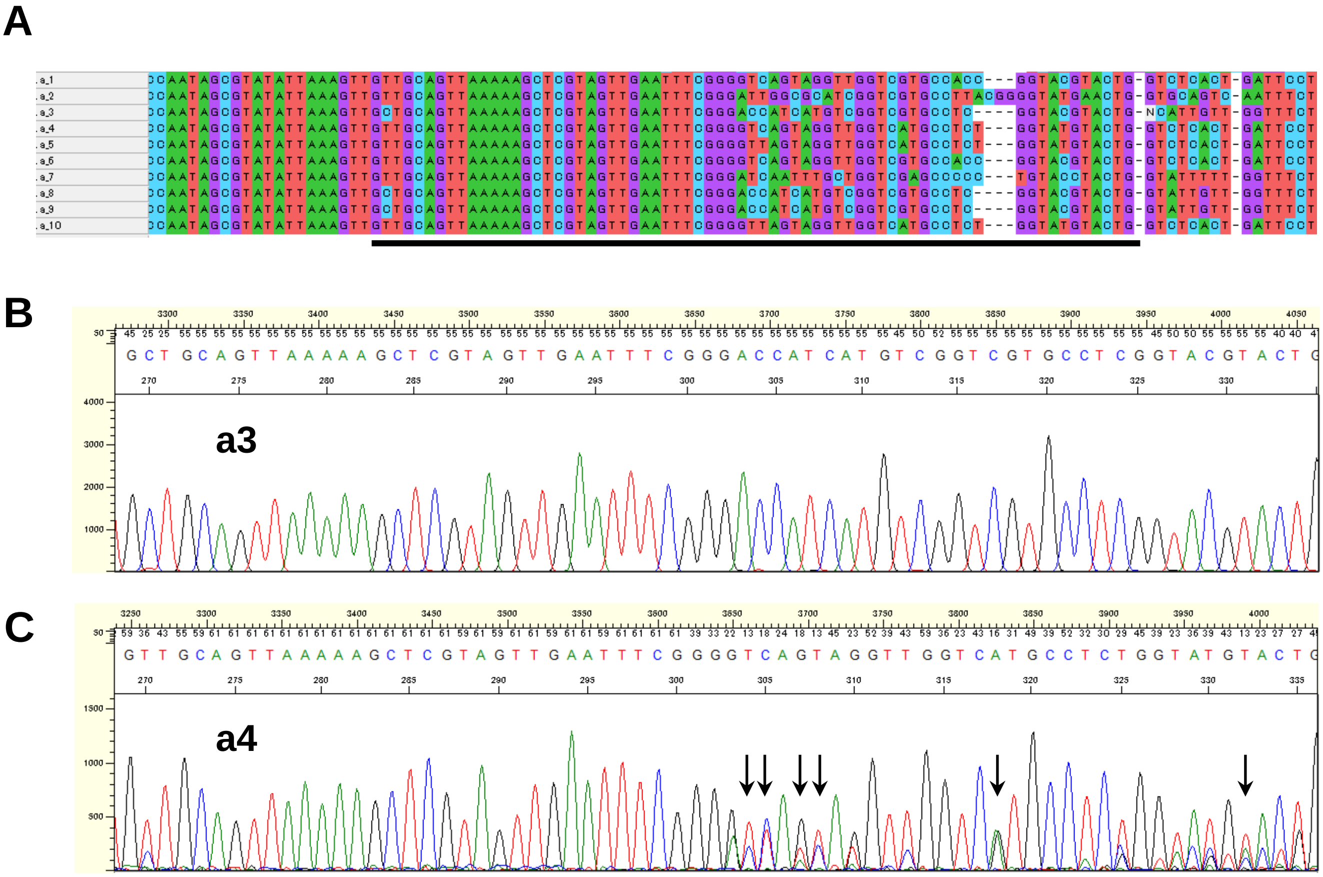

Supplementary data

Figure S4.

Sequence heterogeneity of the mycothalli rDNA Sanger sequences. (**A**) Multiple alignment of first mycothalli rDNA 'meta'-sequences for root a. Multiple sequence alignments were generated using the MUSCLE program on MEGA X software [33]. Sequences corresponding to the nucleotide regions of 2433–2935 (**A**, underlined) or 2433–2935 (**B** and **C**) base pairs of *Rhizophagus irregularis* MUCL43195 (consensus 28) [32] are shown. Arrows in **C** indicate bases with low quality value (QV≤20).
